# Residence in Proximity of an Iron Foundry and Risk of Lung Cancer in the Municipality of Trieste, Italy, 1995–2009

**DOI:** 10.3390/ijerph120809025

**Published:** 2015-07-31

**Authors:** Ettore Bidoli, Fabio Barbone, Paolo Collarile, Francesca Valent, Loris Zanier, Fulvio Daris, Andrea Gini, Silvia Birri, Diego Serraino

**Affiliations:** 1 SOC Epidemiologia e Biostatistica, IRCCS Centro di Riferimento Oncologico di Aviano, 33081 Aviano (PN), Italy; E-Mails: agini@cro.it (A.G.); birris@cro.it (S.B.); serrainod@cro.it (D.S.); 2 Istituto di Igiene ed Epidemiologia clinica, DSMB Università degli Studi di Udine, 33100 Udine, Italy; E-Mail: fabio.barbone@uniud.it; 3 DSM Università degli Studi di Trieste, 34100 Trieste, Italy; 4 SOC Igiene ed Epidemiologia Clinica, Azienda Ospedaliero Universitaria di Udine, 33100 Udine, Italy; 5 Agenzia Regionale Protezione Ambientale, Friuli Venezia Giulia, 33057 Palmanova (UD), Italy; E-Mails: paolo.collarile@arpa.fvg.it (P.C.); fulvio.daris@arpa.fvg.it (F.D.); 6 Direzione Centrale Salute, Friuli Venezia Giulia, Servizio Regionale di Epidemiologia, 33100 Udine, Italy; E-Mails: francesca.valent@regione.fvg.it (F.V.); loris.zanier@regione.fvg.it (L.Z.); 7 Registro Tumori del Friuli Venezia Giulia, 33081 Aviano (PN), Italy

**Keywords:** air pollution, iron foundry, geocoded, lung cancer, northeastern Italy

## Abstract

We assessed the risk of lung cancer in people living near the iron foundry located within the city of Trieste, Northeastern Italy. Between 1995 and 2009, all incident cases of lung cancer and corresponding population were considered. A deposition model of the foundry-specific emissions of SO_2_ defined: “nearby”, “urban”, and “outlying” areas. Rate ratios (RRs) and annual percent changes (APCs) were computed. Among nearby residents, significantly increased risks of lung cancer were noted in men below age 75 years (RR = 1.35 *vs.* urban area; 95% CI: 1.03–1.77). In women, and in men aged 75 years or older, no significant RRs were observed. Conversely, people living in the outlying area appeared to be at lower risk than residents in the urban area- in all age groups, in men (RR = 0.87; 95% CI: 0.78–0.98) and in women (RR = 0.74; 95% CI: 0.62–0.88). Negative statistically significant APC was recorded in men living in urban areas (–2.6%), whereas in women APC significantly increased among those living in the urban area (+2.3%). Multiple interpretations for this observation are plausible, since several factors might have modified and/or confounded the risk of lung cancer, including air pollution from other sources and road traffic, occupational and smoking patterns.

## 1. Introduction

Lung cancer is largely caused by cigarette smoking, while other recognized risk factors are occupational exposures in some industrial facilities [1,2,3,4], air pollution due to road traffic, heating-related and industrial emissions [4,5,6,7,8].

Trieste is a border city located in the northeastern part of Italy, and it is characterized by a major port and by an elevated concentration of industries. Four main historical sources of air pollution are noticeable, *i.e.*, a shipyard, an iron foundry, an incinerator, and urban vehicular traffic [9,10,11]. A case-control study conducted in the early 1990s examined lung cancer mortality in men living in Trieste according to these sources of pollution. Results from this study, adjusted for smoking, occupational exposure to respiratory carcinogens and social class showed a moderate elevation in risk among men residing in all the four polluted areas, with significant variations according to histologic types [10]. A later analysis of these data, which included further geographical modeling of distances from the sources, specified that the risk of lung cancer was especially high among residents in the city center of Trieste or near the incinerator [11]. Additional publications from that case-control study estimated that, in Trieste, the lung cancer attributable risk was 87.5% for cigarette smoking, while it ranged between 16.0% and 23% for occupational exposures [9]. Furthermore, the risk of lung cancer attributable to smoking seemed to be lower for adenocarcinoma than for other histological types [12].

Since 1896, an iron foundry has been located within Trieste city limits, in the Servola District, on 560,000 m^2^ of port land. The plant produces mainly pig iron and it includes the coke ovens, the iron ore agglomeration plant, two blast furnaces and the casting machine. The Regional Agency for Environmental Protection (ARPA-FVG) has identified point and diffuse emissions within the plant. These sources contribute partially to the overall atmospheric concentrations of fine particulate (PM_10_ and PM_2.5_) and gases (NO_2_ and SO_2_) in Trieste. However, local environmental monitoring systems have determined that air pollution in Servola is characterized in particular by diffuse emissions of polycyclic aromatic hydrocarbons (PAHs) from the coke oven of the iron foundry. Several iron foundry processes and products have been classified as carcinogenic to humans by the International Agency for Research on Cancer [13]. Occupational exposure to coke oven processes is associated with a well documented excess of lung cancer risk [14]. In addition to PAHs, environmental pollutants from the coke process include: particulates, other organic compounds, acid gases, benzene, napthalene, and hydrogen sulphide [15].

Since previous studies by Barbone *et al.* [10,11,12] were restricted to men, the potential health effects of long term exposure in women residing near this foundry have never been explored. Thus, we delineated a risk area by means of a deposition modeling of foundry-specific emissions and the whole population of men and women in Trieste was included.

Cancer incidence in Trieste is recorded by the population-based cancer registry of Friuli Venezia Giulia region (CR-FVG). We took advantage of the availability of computerized incidence data recorded by CR-FVG over the 1995–2009 period to describe lung cancer incidence in the whole resident population of Trieste, according to residence at various distances from the coke oven of the iron foundry.

## 2. Experimental Section

### Materials and Methods

All incident cases of lung cancer (2096 men and 955 women) diagnosed during the period 1995–2009 in the resident population of Trieste were extracted from the population-based CR-FVG (http://ci5.iarc.fr/). For the aims of this analysis, incidence data were disentangled by age (quinquennia), sex, year of diagnosis, and histological subtype. The International Classification of Diseases, 10th edition, was used for lung cancer classification (C33–34). The International Classification of Diseases for Oncology, third version (ICDO-3), was used for the classification of histologic subtypes: adenocarcinoma, squamous cell carcinoma, and other and unspecified morphologies. Cases diagnosed at autopsy only (159 men and 109 women) were included in the analyses.

The corresponding structure of the resident population in the Trieste municipality by sex, age (quinquennia), and calendar year (from 1995 to 2009) was obtained from the regional healthcare population database. The geocoded population of this database was linked to the cancer registry data to determine the home address of incident cases of lung cancer. Eighty-two cases of lung cancers (51 men and 31 women) were excluded from the study due to inaccuracy in the home address reported in the database.

An exploratory near-*vs.*-far analysis was conducted to assess the risk of lung cancer according to various residential distances from dispersion models from coke ovens [14]. Three risk areas were defined a priori according to the following criteria: (1) “nearby the foundry” was considered the most likely area of exposure to foundry emissions defined by ARPA-FVG. This area is within 800 m from the foundry, and was delineated by means of a deposition modeling of the foundry-specific emissions of sulfur dioxide (SO_2_). No other major sources of industrial pollution were located within this restricted area; (2) the “urban and industrial” area of Trieste included the city center, the shipyard, and the incinerator. In this area, previous estimates of lung cancer risk in men were published, and it was considered the reference area [10,11]; (3) the “outlying” area, which is largely exempt from major industries, included all the remaining residents of the Trieste municipality. The shortest distance between home residence and the iron foundry is 3.6 km.

Coordinates of lung cancer cases, population, and contours of the three risk areas were imported into Geomedia (v6.1), a geographic information system (GIS). All incident cases and the whole population within each area were thus exported to compute indicators by statistical software. Nearly 7% of the incident cases moved their residence across risk areas in the 15 years before lung cancer diagnosis.

Age-standardized incidence rates (ASRs) per 100,000 (to the Italian population measured at the 2001 national census) (www.istat.it) were calculated for the whole examined period—or for each calendar year from 1995 to 2009—in both sexes and two age groups (<75 and ≥75 years) according to risk area and histological subtypes. ASRs and their corresponding standard errors were calculated using SEER*Stat (Surveillance Research Program, National Cancer Institute SEER*Stat software, seer.cancer.gov/seerstat, version 8.1.2). Rate Ratios (RRs) and 95% confidence intervals (CI) [16] were computed from the ASRs considering the urban area of Trieste as the reference category.

The computation of annual percent change (APC) [17,18] of incidence rates was calculated for the whole 1995–2009 period. APCs were calculated in both sexes and by two age groups (<75 years and ≥75 years) according to risk area. APCs were estimated by fitting a linear regression line to the natural logarithm of annual incidence rates using calendar year as a regressor variable. This calculation assumes that the incidence rates changed at a constant rate over the whole calendar-year interval examined, and the validity of this assumption was checked by merely examining plotted curves. APCs were computed using the Joinpoint software and their statistical significance (*p* < 0.05) was calculated by means of a Student’s t-distribution [18].

## 3. Results

Table 1 reports the number of incident cases of lung cancer, the ASRs, and the RRs according to age, sex and risk areas. A gradient in ASR according to risk area emerged only in men, particularly in those under 75 years of age—from 100.2 cases/100,000/year near the foundry to 65.3 cases/100,000/year in the outlying area (Table 1). Overall, the risk of lung cancer tended to be lower in the outlying area in both men (RR = 0.87; 95% CI: 0.72–0.98) and women (RR = 0.74; 95% CI: 0.62–0.88), than in the urban area. This pattern of risk was particularly evident in women under 75 years of age (RR = 0.65; 95% CI: 0.51–0.85). Among men and women living near the foundry, a statistically significant increased risk of lung cancer was noted solely for men under 75 years of age (RR = 1.35, 95% CI: 1.03–1.77), as compared to those living in the urban area (Table 1).

RRs of lung cancer disentangled by histologic subtype highlighted a significantly increased risk of squamous cell carcinomas for men living near the foundry (RR = 1.57 as compared to those living in the urban area), with a particularly elevated risk in those younger than 75 years (RR = 1.74, 95% CI: 1.05–2.90). Conversely, no significant associations with the risk of lung cancer emerged for women living near the foundry across the various strata of age and morphology (Table 2). A significantly decreased risk in the outlying area was observed in men for adenocarcinomas (RR = 0.76; 95% CI: 0.58–0.99) and in women for other and unspecified morphologies (RR = 0.58; 95% CI: 0.39–0.85). In the ≥75 years age group, a significantly decreased risk, in the outlying area, was displayed by men for other and unspecified morphologies (RR = 0.79; 95% CI: 0.63–1.00) and in women for the squamous cell carcinomas (RR = 0.31; 95% CI: 0.11–0.86) (Table 2).

**Table 1:** Incident cases of lung cancer **^1^**, age-standardized rates on Italian population (ASR-Ita **^2^**) and rate ratios (RR), with corresponding 95% confidence intervals (CI), by sex, and risk area according to age groups during the period 1995–2009 in the municipality of Trieste **^3^**.

Risk Area	<75 Years					≥75 Years					All Ages				
	N	ASR-Ita X100,000	(SE) ^4^	RR	(95% CI)	N	ASR-Ita X100,000	(SE) ^4^	RR	(95% CI)	N	ASR-Ita X100,000	(SE) ^4^	RR	(95% CI)
Men																
Near the foundry (<800 m)	55	100.2	(13.6)	1.35	(1.03–1.77)	22	447.3	(100.9)	0.89	(0.58–1.36)	77	122.0	(14.2)	1.21	(0.96–1.52)
Urban	961	74.1	(2.4)	1		654	502.2	(20.7)	1		1615	100.9	(2.6)	1	
Outlying	249	65.3	(4.1)	0.88	(0.77–1.01)	155	432.7	(36.3)	0.86	(0.72–1.03)	404	88.3	(4.5)	0.87	(0.78–0.98)
Women															
Near the foundry (<800 m)	15	26.8	(7.0)	1.01	(0.60–1.69)	9	111.1	(38.1)	0.69	(0.36–1.34)	24	35.5	(7.4)	0.88	(0.58–1.32)
Urban	368	26.6	(1.4)	1		405	160.8	(8.3)	1		773	40.5	(1.5)	1	
Outlying	69	17.4	(2.1)	0.65	(0.51–0.85)	89	138.3	(15.1)	0.86	(0.68–1.08)	158	29.9	(2.5)	0.74	(0.62–0.88)

Notes: **^1^** Cases diagnosed at autopsy included; **^2^** Italian population at 2001 census; ^**3**^ Data from Friuli Venezia Giulia Cancer registry; **^4^** SE: Standard Error.

**Table 2:** Incident cases of lung cancer **^1^**, age-standardized rates on Italian population (ASR-Ita **^2^**) and rate ratios (RR), with corresponding 95% confidence intervals (CI), by sex, morphology, and risk area according to age groups during the 1995–2009 in the municipality of Trieste **^3^**.

Risk Area	<75 Years						≥75 Years						All Ages					
	N	ASR-Ita X100,000	(SE) ^4^	RR	(95% CI)		N	ASR-Ita X100,000	(SE) ^4^	RR	(95% CI)		N	ASR-Ita X100,000	(SE) ^4^	RR	(95% CI)	
Men																		
Adenocarcinoma																		
Near the foundry (<800 m)	14	25.4	(6.8)	1.14	(0.67–1.94)		2	58.6	(41.4)	0.71	(0.17–2.86)		16	27.4	(6.9)	1.05	(0.64–1.73)	
Urban	287	22.3	(1.3)	1			104	83.0	(8.5)	1			391	26.1	(1.3)	1		
Outlying	64	16.8	(2.1)	0.76	(0.58–0.99)		35	95.4	(16.9)	1.15	(0.78–1.69)		99	21.8	(2.2)	0.83	(0.67–1.04)	
Squamous cell carcinoma																		
Near the foundry (<800 m)	16	29.7	(7.5)	1.74	(1.05–2.90)		6	139.7	(59.3)	1.18	(0.52–2.68)		22	36.6	(7.9)	1.57	(1.02–2.41)	
Urban	222	17.0	(1.1)	1			141	118.1	(10.3)	1			363	23.3	(1.3)	1		
Outlying	66	17.2	(2.1)	1.01	(0.77–1.33)		31	98.2	(18.0)	0.83	(0.56–1.23)		97	22.3	(2.3)	0.95	(0.76–1.19)	
Other and unspecified																		
Near the foundry (<800 m)	25	45.2	(9.1)	1.30	(0.87–1.94)		14	249.0	(70.4)	0.83	(0.49–1.41)		39	58.0	(9.6)	1.13	(0.82–1.55)	
Urban	452	34.8	(1.6)	1			409	301.1	(15.9)	1			861	51.5	(1.8)	1		
Outlying	119	31.2	(2.9)	0.90	(0.73–1.10)		89	239.1	(26.6)	0.79	(0.63–1.00)		208	44.3	(3.2)	0.86	(0.74–1.00)	
Women																		
Adenocarcinoma																		
Near the foundry (<800 m)	7	12.9	(5.0)	1.31	(0.61–2.81)		1	15.5	(15.5)	0.39	(0.05–2.81)		8	13.1	(4.7)	1.02	(0.51–2.07)	
Urban	134	9.8	(0.9)	1			100	39.4	(4.1)	1			234	12.8	(0.9)	1		
Outlying	32	8.1	(1.4)	0.83	(0.56–1.22)		23	37.7	(8.0)	0.96	(0.61–1.50)		55	11.2	(1.5)	0.87	(0.65–1.17)	
Squamous cell carcinoma																		
Near the foundry (<800 m)	2	3.6	(2.6)	1.00	(0.24–4.11)		1	15.5	(15.5)	0.81	(0.11–5.90)		3	4.8	(2.8)	0.93	(0.29–2.94)	
Urban	50	3.6	(0.5)	1			42	19.0	(3.0)	1			92	5.2	(0.6)	1		
Outlying	7	1.7	(0.6)	0.46	(0.21–1.01)		4	5.8	(3.0)	0.31	(0.11–0.86)		11	2.1	(0.6)	0.40	(0.21–0.75)	
Other and unspecified																		
Near the foundry (<800 m)	6	10.3	(4.2)	0.78	(0.35–1.76)		7	80.2	(31.2)	0.78	(0.37–1.66)		13	17.5	(5.0)	0.78	(0.45–1.36)	
Urban	184	13.2	(1.0)	1			263	102.4	(6.6)	1			447	22.4	(1.1)	1		
Outlying	30	7.6	(1.4)	0.58	(0.39–0.85)		62	94.8	(12.5)	0.93	(0.70–1.22)		92	16.6	(1.8)	0.74	(0.59–0.93)	

Notes: **^1^** Cases diagnosed at autopsy included; **^2^** Italian population at 2001 census; **^3^** Data from Friuli Venezia Giulia Cancer registry; **^4^** SE: Standard Error.

APCs (% per year) of lung cancer incidence rates for the period 1995–2009 are reported in Table 3. Among men living in the urban area, a significantly negative APC emerged (APC = −3.2% per year), whereas the inverse occurred for women (APC = +2.3% per year). Men under 75 years of age showed a significantly negative APCs in urban area (APC = −3.2% per year; 95% CI: −4.9; −1.5) and in outlying area (APC = −2.8%; 95% CI: −5.5; −0.1). Although not significant, a decrease was observed in the area near the foundry (APC = −1.9%; 95% CI: −6.8; +3.3). This favorable temporal pattern was not observed in the ≥75-year age group.

**Table 3:** Annual percent changes (APC), and corresponding 95% confidence intervals (CI), of lung cancer **^1^** incidence by sex, and risk area according to age groups during the period 1995–2009 in the municipality of Trieste **^2^**.

Risk Area		<75 Years			≥75 Years		All Ages		
		APC	(95% CI)		APC	(95% CI)		APC	(95% CI)
Men									
Near the foundry (<800 m)		−1.9	(−6.8; +3.3)		−0.1	(−6.8; +7.1)		−2.2	(−7.4; +3.4)
Urban		−3.2 *	(−4.9; −1.5)		−1.2	(−3.7; +1.3)		−2.6 *	(−3.9; −1.3)
Outlying		−2.8 *	(−5.5; −0.1)		−2.8	(−7.0; +1.6)		−1.9	(−4.6; +1.0)
Women									
Near the foundry (<800 m)		+6.5	(−5.3; +19.7)		−6.4	(−21.8; +12.0)		0.0	(−10.9; +12.3)
Urban		+1.9	(−1.1; +5.0)		+2.5 *	(+0.0; +5.1)		+2.3 *	(+0.1; +4.4)
Outlying		−1.4	(−6.6; +4.1)		−1.6	(−8.4; +5.7)		−1.5	(−6.3; +3.4)

Notes: **^1^** Cases diagnosed at autopsy included; **^2^** Data from Friuli Venezia Giulia Cancer registry; * *p* < 0.05.

In women, some variability in temporal trends was observed. This variability is partially compatible with random variation due to a small number of incident cases in the risk areas. In particular, women under 75 years of age displayed a positive APC, though not statistically significant, near the foundry (APC = +6.5%; 95% CI: −5.3; +19.7), whereas, above 75 years of age, a statistically significant positive APC was observed in the urban area (APC = +2.5%; 95% CI: +0.0; +5.1).

## 4. Discussion

In this population-based study conducted in the city of Trieste, Northeastern Italy, we investigated the risk of lung cancer according to the distance of the residence from an iron foundry. An excess risk of lung cancer was observed in men living near the foundry, particularly in those under 75 years of age at lung cancer at diagnosis, and risk was restricted to the squamous cell carcinoma type. Conversely, the risk was close to unity in the women overall, and no excess risk was observed in men or in women older than 75 years. Interestingly, a much lower risk of lung cancer was seen in people of both sexes living in the outlying area. Finally, between 1995 and 2009, in men incidence rates decreased in all the areas examined, whereas women displayed variability in temporal trends depending on area and age at diagnosis.

The observed discrepancy in the geographical pattern between men and women does not support the hypothesis that residence near the iron foundry of Trieste is associated with an excess of risk of lung cancer incidence due to air pollution. Nonetheless, the excess risk in men is intriguing. The different risks between sexes, the excess of squamous cell carcinomas in men living near the foundry, and the decreasing rates in men matched to the slight variation of rates in women, suggest that etiological factors other than air pollution might have played a substantial role in modifying the risk of lung cancer in this population. Since working in an iron foundry is a known risk factor for lung cancer [13], and prevalence of cigarette smoking is changing between sexes in Italy [19] it seems likely, although not demonstrable by this observational study, that the excess of lung cancer cases recorded in men may be attributable to a mixture of factors, including occupational exposures, air pollution due to sources other than the iron foundry, and smoking.

Findings from epidemiological investigations in high risk areas near iron foundries are inconsistent. Two analytical studies [20,21] that adjusted for smoking and a descriptive study [22] that adjusted for socioeconomic status showed that living near an iron foundry was not a risk factor for lung cancer incidence or mortality. Descriptive studies that did not adjust for smoking displayed an increased risk of lung cancer living near iron foundries [23,24,25]. In addition, smoking and occupational exposure may play a more specific role in the etiology of squamous cell carcinoma and small cell cancers than with adenocarcinoma [26]. Finally, a case-control study conducted in the early 1990s in Trieste [10], adjusted for smoking, occupation, and social class, showed that living in the urban area or near one of the three industrial sources of pollution (an incinerator, a shipyard and the above mentioned iron foundry) was indeed associated with an increased risk of lung cancer in men. That study was based not only on distance from pollution sources but also on objective, environmental measurements of air pollution. Conclusions from that study involved the identification of multiple industrial and urban air pollution sources as determinants of lung cancer among men in Trieste.

Some limitations of this descriptive investigation are common to other observational studies, namely the lack of individual assessment of environmental exposure and its long-term effects by means of aggregated data, and the lack of information on smoking, the most relevant confounder. This variable was not included in the analysis, since no information on this habit was reported in the regional healthcare database. However, we sought to minimize this problem by performing separate analyses by sex, and age group to roughly define subgroups with different proportions of smokers. Clearly, this adjustment is only indirect, and there is thus a high likelihood of our results having been influenced by tobacco-related factors. Secondly, sex-specific occupational exposures may have also influenced the different risks between men and women, but occupational history was not available. Thirdly, analyses by subgroups with small number of incident lung cancer cases (e.g., histology subtype or women) should be approached with care due to wide random variability. Fourthly, no information was available about the daily time spent in each risk area and of the effect of potential sex-specific differences. Finally, although the presence of other industries in the urban area of Trieste may render interpretation of results difficult with regard to the role of a single source of air pollution, no other major sources of industrial pollution existed in the area near foundry.

Several strengths of this investigation deserve attention. As environmental exposure is concerned, our measures were based on the residential location of the participants, and, despite the fact that they could only take into account the geographical coordinates of the last reported residence, our study population was relatively stable, with nearly 93% of lung cancer cases living in their risk area of residence for more than 15 years. It should be noted that the lung cancer cases and the general population were derived from two regional databases (CR-FVG and healthcare population), which provide high-quality data and guarantee the complete coverage of the resident population. In particular, the CR-FVG is listed in the Cancer in Five Continents publications [27].

## 5. Conclusions

This observational study showed that residing within 800 meters from the coke oven of an iron foundry was associated with lung cancer risk in men aged <75, but not in women. Other factors might have modified and/or confounded the risk of lung cancer, including air pollution from other sources and occupational and smoking patterns.

## References

[1] Rodríguez V., Tardón A., Kogevinas M., Prieto C.S., Cueto A., García M., Menéndez I.A., Zaplana J. (2000). Lung cancer risk in iron and steel foundry workers: A nested case control study in Asturias, Spain. Amer. J. Ind. Med..

[2] Whitrow M.J., Smith B.J., Pilotto L.S., Pisaniello D., Nitschke M. (2003). Environmental exposure to carcinogens causing lung cancer: Epidemiological evidence from the medical literature. Respirology.

[3] Hoshuyama T., Pan G., Tanaka C., Feng Y., Yu L., Liu T., Liu L., Hanaoka T., Takahashi K. (2006). Mortality of iron-steel workers in Anshan, China: A retrospective cohort study. Int. J. Occup. Environ. Health.

[4] Field R.W., Withers B.L. (2012). Occupational and environmental causes of lung cancer. Clin. Chest Med..

[5] Vena J.E. (1982). Air pollution as a risk factor in lung cancer. Amer. J. Epidemiol..

[6] Cohen A.J. (2000). Outdoor air pollution and lung cancer. Environ. Health Perspect..

[7] Nyberg F., Gustavsson P., Jarup L., Bellander T., Berglind N., Jakobsson R., Pershagen G. (2000). Urban air pollution and lung cancer in Stockholm. Epidemiology.

[8] International Agency for Research on Cancer (2013). Monographs on the Evaluation of the Carcinogenic Risk of Chemicals to Humans. Diesel and Gasoline Engine Exhausts and Some Nitroarenes.

[9] Bovenzi M., Stanta G., Antiga G., Peruzzo P., Cavallieri F. (1993). Occupational exposure and lung cancer risk in a coastal area of northeastern Italy. Int. Arch. Occup. Environ. Health.

[10] Barbone F., Bovenzi M., Cavallieri F., Stanta G. (1995). Air pollution and lung cancer in Trieste, Italy. Amer. J. Epidemiol..

[11] Biggeri A., Barbone F., Lagazio C., Bovenzi M., Stanta G. (1996). Air pollution and lung cancer in Trieste, Italy: spatial analysis of risk as a function of distance from sources. Environ. Health Perspect..

[12] Barbone F., Bovenzi M., Cavallieri F., Stanta G. (1997). Cigarette smoking and histologic type of lung cancer in men. Chest.

[13] International Agency for Research on Cancer (1984). Polynuclear aromatic compounds, part 3, industrial exposures in aluminum production, coal gasification, coke production, and iron and steel founding. Monographs on the Evaluation of the Carcinogenic Risk of Chemicals to Humans.

[14] (2005). US-EPA. http://www.epa.gov/scram001/guidance/guide/appw_05.pdf.

[15] (2008). US-EPA. http://www.epa.gov/ttnchie1/ap42/ch12/final/c12s02_may08.pdf.

[16] Rothman K.J., Greenland S., Lash T.L. (2008). Modern Epidemiology.

[17] Kim H.J., Fay M.P., Feuer E.J., Midthune D.N. (2000). Permutation tests for joinpoint regression with applications to cancer rates. Stat. Med..

[18] National Cancer Institute, Joinpoint Regression Program. http://www.srab.cancer.gov/jointpoint.

[19] La Sorveglianza Passi. http://www.epicentro.iss.it/passi/.

[20] Brown L.M., Pottern L.M., Blot W.J. (1984). Lung cancer in relation to environmental pollutants emitted from industrial sources. Environ. Res..

[21] Jöckel K.H., Ahrens W., Wichmann H.E., Becher H., Bolm-Audorff U., Jahn I., Molik B., Greiser E., Timm J. (1992). Occupational and environmental hazards associated with lung cancer. Int. J. Epidemiol..

[22] Breugelmans O., Ameling C., Marra M., Fischer P., van de Kassteele J., Lijzen J., Oosterlee A., Keuken R., Visser O., Houthuijs D., van Wiechen C. (2013). Lung cancer risk and past exposure to emissions from a large steel plant. J. Environ. Public Health.

[23] Lloyd O.L. (1978). Respiratory-cancer clustering associated with localised industrial air pollution. Lancet.

[24] Lloyd O.L., Smith G., Lloyd M.M., Holland Y., Gailey F. (1985). Raised mortality from lung cancer and high sex ratios of births associated with industrial pollution. Brit. J. Ind. Med..

[25] Smith G.H., Williams F.L., Lloyd O.L. (1987). Respiratory cancer and air pollution from iron foundries in a Scottish town: An epidemiological and environmental study. Brit. J. Ind. Med..

[26] Lubin J.H., Blot W.J. (1984). Assessment of lung cancer risk factors by histologic category. J. Natl. Cancer Inst..

[27] Cancer in Five Continents. http://ci5.iarc.fr/.

